# The value of incorporating patient-consulted medication reconciliation in influencing drug-related actions in the outpatient rheumatology setting

**DOI:** 10.1186/s12913-022-08391-7

**Published:** 2022-08-05

**Authors:** Denise J. van der Nat, Victor J. B. Huiskes, Aatke van der Maas, Judith Y. M. N. Derijks-Engwegen, Hein A. W. van Onzenoort, Bart J. F. van den Bemt

**Affiliations:** 1 Department of Clinical Pharmacy, Amphia Hospital, Breda, the Netherlands; 2 Department of Pharmacy, St. Maartenskliniek, Nijmegen, the Netherlands; 3 Department of Pharmacy, Radboud Institute for Health Sciences (RIHS), Radboud University Medical Centre, Nijmegen, the Netherlands; 4 Department of Rheumatology, St. Maartenskliniek, Nijmegen, the Netherlands; 5 Clinical Pharmacy and Toxicology, Maastricht University Medical Center+, Maastricht, the Netherlands

**Keywords:** Medication reconciliation, Drug information, Medication safety, Outpatient clinic

## Abstract

**Background:**

Unintentional changes to patients’ medicine regimens and drug non-adherence are discovered by medication reconciliation. High numbers of outpatient visits and medication reconciliation being time-consuming, make it challenging to perform medication reconciliation for all outpatients. Therefore, we aimed to get insight into the proportion of outpatient visits in which information obtained with medication reconciliation led to additional drug-related actions.

**Methods:**

In October and November 2018, we performed a cross-sectional observational study at the rheumatology outpatient clinic. Based on a standardized data collection form, outpatient visits were observed by a pharmacy technician trained to observe and report all drug-related actions made by the rheumatologist. Afterwards, the nine observed rheumatologists and an expert panel, consisting of two rheumatologists and two pharmacists, were individually asked which drug information reported on the drug list composed by medication reconciliation was required to perform the drug-related actions. The four members of the expert panel discussed until consensus was reached about their assessment of the required information. Subsequently, a researcher determined if the required information was available in digital sources: electronic medical record (electronic prescribing system plus physician’s medical notes) or Dutch Nationwide Medication Record System.

**Results:**

Of the 114 selected patients, 83 (73%) patients were included. If both digital drug sources were available, patient’s input during medication reconciliation resulted in additional information to perform drug-related actions according to the rheumatologist in 0% of the visits and according to the expert panel in 14%. If there was only access to the electronic medical record, the proportions were 8 and 29%, respectively. Patient’s input was especially required for starting a new drug and discussing drug-related problems.

**Conclusions:**

If rheumatologists only had access to the electronic medical record, in 1 out of 3 visits the patient provided additional information during medication reconciliation which was required to perform a drug-related action. When rheumatologists had access to two digital sources, patient’s additional input during medication reconciliation was at most 14%. As the added value of patient’s input was highest when rheumatologists prescribe a new drug and/or discuss a drug-related problem, it may be considered that rheumatologists only perform medication reconciliation during the visit when performing one of these actions.

**Supplementary Information:**

The online version contains supplementary material available at 10.1186/s12913-022-08391-7.

## Background

Transitions in healthcare are associated with a high risk of medication errors [1–6]. These errors are often caused by medication discrepancies between patient’s actual medication use and the medication that is listed in a patient’s medical record. Although not all medication discrepancies are clinically relevant, some medication discrepancies result in drug interactions, therapeutic duplication, and other unintended adverse events [2, 7, 8]. If not recognized early, these medication discrepancies will lead to an increased risk of hospital readmissions, emergency room visits and prolonged hospital stay [1, 8–12].

Up to 100% of the patients admitted to a hospital have at least one medication discrepancy [13, 14], of which half of these have the potential to harm patients [15–18]. In the outpatient setting, more than half of the patients visiting the outpatient clinic have at least one medication discrepancy [19–21]. Although the proportion of outpatients with a medication discrepancy is lower, the large number of patients visiting the outpatient clinics results in a high number of patients with at least one medication discrepancy. Because of this, it is important to identify outpatients at risk and prevent harm to those patients.

Medication reconciliation (MR) has been proven as an effective method to reduce medication discrepancies. Most studies demonstrate that MR approximately halves the number of medication discrepancies both in the inpatient and outpatient setting [9, 20, 22, 23]. Besides reducing medication discrepancies, MR has the ability to identify medication non-adherence, low medication literacy, and use of non-prescribed medications such as supplements and vitamins [24]. During MR, the best possible medication history (BPMH) is composed according to the standard operating procedure of the High 5 s project of the World Health Organization [24]. The World Health Organization defined the BPMH as “a medication history obtained by a clinician which includes a thorough history of all regular medication use (prescribed and non-prescribed), using a number of different sources of information” [24]. Drug information sources that are usually available in the Netherlands for MR are the electronical medical record (EMR) and the Nationwide Medication Record System (NMRS) (Table 1). To obtain the BPMH in this setting, pharmacy technicians combine the information provided by a structured patient interview, information from the EMR and information from the NMRS. Inpatients are assessed in the first 24 hours of their admission [28]. This is important because studies shows that Dutch inpatients have on average three medication discrepancies [27, 29, 30]. Also, in the outpatient setting about 83% of the patients have at least one medication discrepancy [20]. However, outpatient MR, such as for the outpatient rheumatology clinic, is not implemented at all Dutch hospitals. At patient’s discharge, all drug changes are communicated back to the patient’s community pharmacy and general practitioner [28].

**Table 1:** Drug information sources

• **Nationwide Medication Record System (NMRS)**. The NMRS is a digital nationwide network which exchanges medication dispensing data from all pharmacies in the Netherlands (provided that the patient has given permission for this data to be shared) [25, 26]. The overview of the NMRS showed all dispended drugs of the past 14 months, including stopped medication.	
• **Electronical medical record (EMR).** The EMR contains (drug) data from patients, as well in the electronic prescribing system as in the physician’s medical notes. Keeping this drug information up to date is the responsibility of the physician.	
• **Patient*.*** Patient’s input was initially obtained by using an online personal health record, in which they checked their pre-filled drug list and were able to add and/or remove drugs [27]. If patients did not use the online personal health record to check their drug list at home, a pharmacy technician interviewed those patients by telephone.	

According to the World Health Organization, MR is classified as one of the top five strategies to ensure patient safety and it is recommended or even mandated by several guidelines and accreditation standards [24, 28, 31, 32]. However, given the time spent and cost to perform MR with each patient (up to 30 minutes) and the large number of patients visiting the outpatient clinic, healthcare organizations struggle to implement MR, especially in settings with a high patient turn-over such as the outpatient clinic [33–35]. Because of this, MR should be targeted to outpatients with a high risk of medication discrepancies that affect the clinical decisions made by the prescriber.

To achieve a more targeted and consequently efficient implementation of MR, it can be hypothesized that for some (low risk) patients MR based on only digital sources might be sufficient, whereas for some (high risk) patients information from the patient in addition to the digital information is essential for the prescriber to perform drug related actions at the outpatient setting. However, no information is available in the literature how often patient reported information during MR leads to other clinical decisions compared to a situation in which only digital information was available. Therefore, the primary aim of this study is to determine the proportion of outpatient visits in which information obtained with MR led to additional drug-related actions.

## Methods

### Setting

A cross-sectional observational study was conducted at the outpatient rheumatology clinic at the Sint Maartenskliniek, Nijmegen, the Netherlands. During this study, outpatient visits were observed in the period between October 31 and November 29, 2018.

### Ethical considerations

The study (file number: 2021–13,272) was approved by the Medical Research Ethics Committee of Arnhem-Nijmegen, the Netherlands. Informed consent was obtained (by the observed rheumatologists) from all individual participants included in the study.

### Selection of observation days and healthcare practitioners

A sample of consultations was taken by selection of consultations with various rheumatologists or physician assistants on as many different dayparts (morning or afternoon) of the week as possible. There is no difference in patient characteristics and/or type of consultations per part of the day and/or practitioner. The selection of the observation dayparts and practitioners was performed by a medical secretary who was not part of the research team and all practitioners were eligible. All consecutive visits (if patients agreed to participate) of the selected practitioner were observed and each selected practitioner was observed for a maximum of one daypart.

### Participants

All consecutive patients visiting the selected rheumatologist on an observation day were eligible. Inclusion criteria were age ≥ 18 years, able to speak and understand the Dutch language and that MR had been performed in the 2 weeks prior to the outpatient visit. Patients were included after obtaining verbal consent. Patients were excluded when MR was not performed prior to the visit or when the rheumatologist requested not to observe a specific patient (for example if the patient was familiar to the observer or was nervous).

### Study design

**Fig. 1:**
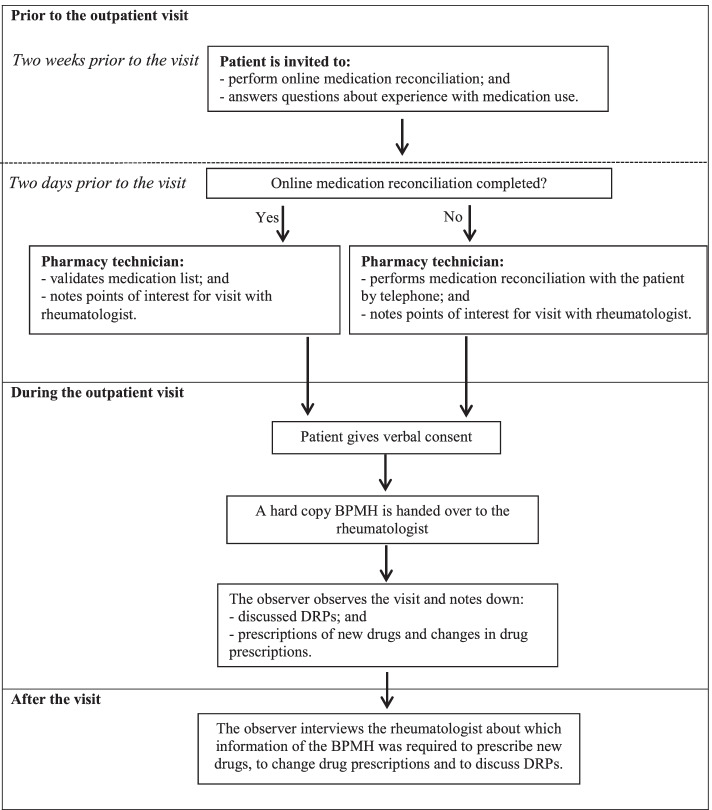
Schematic representation of the study design. *Abbreviations: BPMH, best possible medication history; DRPs, drug-related problems*

A schematic representation of the study design is depicted in Fig. 1.

#### Prior to the outpatient visit

Fourteen to 3 days before the visit, MR was performed with the outpatient by telephone or online using a personal health record. During MR, the BPMH was created based on patient’s input and drug information derived from digital sources (the EMR and NMRS).

#### Observation during the visits

Each visit was observed by a single pharmacy technician. The nine rheumatologists who conducted the outpatient visits were not blinded about the aim of the study. The pharmacy technician was trained to observe and report all drug-related actions made by the rheumatologist, based on a standardized observation scheme and data collection form (see Additional file 1). Drug-related actions performed during an outpatient visit covered changing drugs and discuss drug-related problems (DRPs), defined as events or circumstances involving drug therapies that actually or potentially interfere with an optimal outcome of medical care [36].

#### Measurements directly after the visits: assessment by the rheumatologists

After each outpatient visit, the rheumatologist was interviewed about which information of the BPMH (the presence of a drug, drug strength and/or drug frequency) he/she had used to perform each of the drug-related actions. The rheumatologists did not have the opportunity to discuss the required information with the other rheumatologists. Subsequently, a researcher determined if this required information was also available in the digital sources.

#### Assessment by the expert panel

In 2020, an expert panel consisting of two (of the nine observed) rheumatologists and two pharmacists scored which drug-related information was necessary for the drug-related actions that had been performed during the visits. This was done to get insight into inter-individual differences between rheumatologists about the required drug information to perform the drug-related actions and to get insight into potential underreporting of required drug information caused by time constraints during the interviews after the visits.

First, the members of the expert panel (VH, BvdB, AvdM and JvZ) were individually asked to construct a generic assessment form (see Additional file 2) to consistently define which information is essential to perform 14 observed types of drug-related actions. In 9 of the 14 types of drug-related actions consensus was reached (Additional file 3). Based on the created assessment form, one researcher (DN) scored for all individual drug-related actions which belong to those 9 types of drug related-actions, which information of the BPMH was required to perform these actions. For the remaining 5 of the 14 types of drug-related actions with could not be assessed with the generic assessment form, the individual drug-related actions belonging to those 5 types of drug-related actions were assessed by two members of the expert panel (VH and AvdM). In case of disagreement about the required drug-information for an individual drug-related action, the case was discussed with a third member of the expert panel (BvdB) until consensus was achieved. Finally, a researcher (DN) determined if the required information was also available in the digital sources.

### Outcome measures

The primary outcome of the study was the proportion of outpatient visits in which the patient provided essential drug information in addition to the existing digital information (including the EMR and NMRS) during MR that was required for the rheumatologist to perform drug-related actions. The secondary endpoint was the proportion of outpatient visits in which the patient provided essential information during MR that was not available in the EMR only.

### Data collection

Patients’ drug information was collected from three sources: the NMRS, the EMR and the patient (Table 1). The drug information of the EMR and the NMRS were combined to reflect the situation that both information sources were available for the rheumatologist. Besides that, the BPMH was created based on drug information obtained from the patient and information derived from the EMR and the NMRS.

Drug-related actions performed by the rheumatologists were collected during the visits by the observer. A change in a drug prescription was defined as a change in one of a patient’s drugs as compared to the BPMH. Drug changes were categorized into the following types: start a drug, stop a drug, adjust the dose or frequency of a current drug [28]. DRPs (e.g. experiencing adverse drug reactions, worrying about (potential adverse events of) a drug or using drug in a different way than prescribed), were classified using the DOCUMENT classification system, with modifications as described by Kwint et al. [36, 37]. DRPs were classified independently by two researchers (VH and BvdB).

### Statistical analysis

Three steps were performed to determine the proportion of outpatient visits in which the patient provided essential additional information during MR compared to the digital sources. First, for each drug-related action the researcher determined if the required drug information was available in the EMR, NMRS and BPMH. Second, the number of outpatient visits for which all required drug information was only available in the BPMH (and not in EMR and NMRS) were counted. Third, the proportion of outpatient visits in which patient’s input provided additional essential information compared to the EMR (plus NMRS) was determined.

Data were analysed using IBM SPSS Statistics software version 25. Additional descriptive statistics were provided using mean (± standard deviation [SD]) or median (interquartile range [IQR]) values depending on the (non-)parametric distribution of measured variables.

## Results

### Study population

**Fig. 2:**
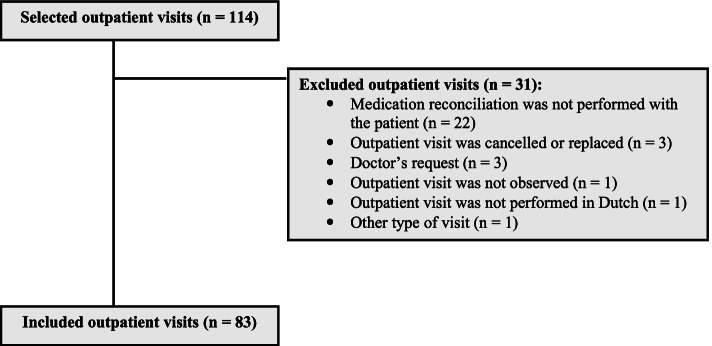
Flowchart of study population. The flowchart displays the reasons of exclusion of the quasi-random selected outpatient visits. At the end of the study 83 outpatient visits were included. *Abbreviations: BPMH, best possible medication history; DRPs, drug-related problems*

Of the 114 selected patients, 83 (73%) patients with a single visit to the outpatient rheumatology clinic were included (Table 2). The visits were performed by nine rheumatologists. The most common reason of exclusion (71%) was that MR was not performed prior to the visit (due to capacity problems of the pharmacy technician team) (Fig. 2). In the study population, 78 (94%) patients had at least one medication discrepancy between the drug list generated with MR and the NMRS plus EMR. Compared to the EMR alone, 68 (82%) patients had at least one medication discrepancy.

**Table 2:** Characteristics of the study sample (*n* = 83)

**Characteristic**	
**Age (years, mean (SD))**	57.2 (15.6)
**Men, N (%)**	33 (39.8)
**Number of drugs on the BPMH, median (IQR)**	6.0 (3.0–9.0)
**Number of rheumatology-related drugs on the BPMH, median (IQR)**	3.0 (2.0–4.0)
**Type of visit, N (%)**	
First visit	13 (15.7)
Follow-up appointment	70 (84.3)
**Days since previous outpatient visit, median (IQR)**	85 (20–183)
**MR performed for the first time, N (%)**	20 (24.1)
**Days since previous MR, median (IQR)**	143 (69–210)
**Person who performed MR, N (%)**	
Patient using a personal health record	37 (44.6)
Pharmacy technician	46 (55.4)
**Type of prescriber, N (%)**	
Physician assistant	8 (9.6)
Rheumatologist	75 (90.4)

*Abbreviations*: *BPMH* Best possible medication history, *MR* Medication reconciliation, *IQR* Interquartile range, *SD* Standard deviation

### Performed drug-related actions

Rheumatologists performed at least one drug-related action in 65 (78%) of the outpatient visits (Table 3). In total, 123 drug-related actions were performed by the rheumatologists, 67 (54%) of the drug-related actions concerned a change of drugs and 56 (46%) drug-related actions concerned the discussion of DRPs (Table 4). In 33 (51%) of the outpatient visits in which a drug-related action was performed, the rheumatologist indicated that he/she needed drug information to perform these drug-related actions. According to the expert panel, in 59 (91%) of the outpatient visits in which a drug-related action was performed, drug information was required to perform the drug-related actions (Table 3).

**Table 3:** Proportion of outpatient visits (*n* = 83) with additional essential information provided by the patient during medication reconciliation. In 65 (78%) of the outpatient visits a drug-related action was performed. The outpatient visits were sorted by the type of drug-related action performed during the visit. The proportions were determined based on the required drug information indicated by the rheumatologists and expert panel. The proportions in bold indicate the proportion of visits in which the patient provided additional essential information during MR compared to the EMR (plus NMRS) examined in the total study sample (*n =* 83)

Type of drug related action performed during the visit	Number of visits with requirement of drug information to perform certain type of action, N (%)		Proportion of visits in which the patient provided additional essential information during MR compared to:			
			EMR, N (%)		EMR plus NMRS, N (%)	
	Rheumatologists	Expert panel	Rheumatologist	Expert panel	Rheumatologist	Expert panel
**Change drugs (*n =*** **24)**	20 (83)	24 (100)	7 (35)	9 (38)	0 (0)	1 (4)
**Discuss DRPs (*n =*** **17)**	8 (47)	15 (88)	0 (0)	11 (73)	0 (0)	9 (60)
**Change drugs and discuss DRPs (*n =*** **29)**	5 (21)	20 (83)	0 (0)	4 (20)	0 (0)	2 (10)
***Perform all drug-related actions (n*** **=** ***65)***	33 (51)	59 (91)	7 (21)	24 (41)	0 (0)	12 (20)
***Total number of visits (*n =** ***83)***	**33 (40)**	**59 (71)**	**7 (8)**	**24 (29)**	**0 (0)**	**12 (14)**

*Abbreviations*: *DRP* Drug related problem, *EMR* Electronic medical record, *MR* Medication reconciliation, *NMRS* Nationwide Medication Record System

**Table 4:** Type of drug-related actions where the patient provided additional essential information during medication reconciliation (MR). The table shows the proportions of outpatient visits (*n =* 83) with additional essential information provided by the patient during MR sorted by the type of drug-related action. The proportions were determined based on the required drug information indicated by the rheumatologists and expert panel

Type of drug-related action	Number of visits with requirement of drug information for certain type of action		Proportion of visits in which patient’s interview during MR provided additional essential information available compared to:			
			EMR, N (%)		EMR plus NMRS, N (%)	
	Rheumatologists	Expert panel	Rheumatologists	Expert panel	Rheumatologists	Expert panel
**Drug changes**						
Start a drug (*n =* 24)	8	13	3 (38)	7 (54)	0 (0)	1 (8)
Stop a drug (*n =* 23)	11	22	3 (27)	4 (18)	0 (0)	0 (0)
Change frequency of current drug (*n =* 13)	6	13	1 (17)	3 (23)	0 (0)	2 (15)
Change strength of current drug (*n =* 6)	3	6	0 (0)	0 (0)	0 (0)	0 (0)
Change dosage form of current drug (*n =* 1)	1	1	0 (0)	0 (0)	0 (0)	0 (0)
***Total drug changes (n*** **=** ***67)***						
**'DRPs**						
Drug selection (*n =* 19)	4	14	0 (0)	7 (50)	0 (0)	5 (36)
Dosing problems (*n =* 1)	1	1	0 (0)	0 (0)	0 (0)	0 (0)
Compliance (*n =* 5)	1	2	0 (0)	1 (50)	0 (0)	0 (0)
Untreated indications (*n =* 0)	0	0	0 (0)	0 (0)	0 (0)	0 (0)
Monitoring (*n =* 1)	1	1	0 (0)	1 (100)	0 (0)	1 (100)
Education or information (*n =* 8)	0	6	0 (0)	1 (17)	0 (0)	1 (100)
Non-clinical (*n =* 0)	0	0	0 (0)	0 (0)	0 (0)	0 (0)
Toxicity or adverse reaction *n =* 22)	6	17	0 (0)	0 (0)	0 (0)	0 (0)
***Total DRPs (n*** **=** ***56)***						
**Total drug changes and DRPs (*n =*** **123)**						

*Abbreviations*: *DRPs* Drug-related problems, *EMR* Electronical medical record, *MR* Medication reconciliation, *NMRS* Nationwide Medication Record System

### Primary outcome: proportion of visits with additional essential information provided by medication reconciliation

According to the individual rheumatologists, in none of the outpatient visits the patient provided additional information during MR required for the rheumatologists to perform drug-related actions that was not available in the EMR plus NMRS. According to the expert panel this was the case in 14% of the visits. Compared to the EMR alone, patient’s input during MR resulted in additional essential information according to the rheumatologists in 8% and according to the expert panel in 29% of the outpatient visits (Table 3). For example, in one case the rheumatologist stopped etoricoxib. To perform this drug-related action, it is relevant to know that the patient used etoricoxib. However, use of etoricoxib was not recorded in the EMR, whereas the patient did provide this information during MR. In another case, the rheumatologist started celecoxib. For this, the use of other pain medication and a proton pump inhibitor is relevant. Use of paracetamol, etoricoxib, codeine and pantoprazole was not reported in the EMR, but was provided by the patient during MR.

We observed that the added value of patient’s input during MR depended on the type of performed drug-related action (Table 4). According to the expert panel, the additional information – compared to the EMR and NMRS – provided by the patient, concerned information needed to start a new drug, to change the frequency of a current drug and to discuss DRPs about drug selection, education and monitoring.

## Discussion

To our knowledge, this is the first study examining the need of MR for prescribing drugs in an outpatient rheumatology clinic. We examined if the patient provided additional essential information during MR that was not available in digital sources, required for rheumatologists to perform drug-related actions during outpatient visits. According to the rheumatologists, patients provided additional essential information during MR in 8 and 0% of the outpatient visits if compared to the EMR and the EMR plus NMRS, respectively. According to the expert panel, these proportions were 29 and 14%.

Studies on the added value of MR in the outpatient setting are lacking. Until now, studies in the outpatient clinics have only examined the effect of MR on the identification and reduction of MDs, without taking into account the value of the additional information provided by the patient during MR [20, 23, 38, 39]. According to the guidelines, it is necessary to perform MR prior to or during the prescribing of drugs [24, 31]. Our results indicate the opposite for the outpatient rheumatology setting when rheumatologists had access to two digital drug sources (EMR and NMRS): patient’s input during MR resulted in additional essential information according to the rheumatologist in 0% and according to the expert panel in 14% of the outpatient visits. Although the proportion of patients with at least one medication discrepancy was high in our research (94%), it seems not efficient to obtain patient’s input prior to all outpatient visits. Based on this, we assume that information from identified medication discrepancies is only required to perform drug-related actions in a few cases.

Furthermore, we observed that the added value of patient’s input during MR depended on the type of performed drug-related action. According to both the rheumatologists and the expert panel, in about half of the cases the additional essential information provided by the patient was required to start a new drug. This may be explained by the fact that starting new drugs requires information about patient’s current use of both rheumatism-related and non-rheumatism-related drugs, which may not be completely reported in the EMR of rheumatologists. This is supported by other studies demonstrating that performing MR is relevant to prevent drug-drug interactions, which can arise when a new drug is started [40–42]. Also discussing DRPs may require information about non-rheumatism-related drugs. This was confirmed by the observation that the patient provided additional essential information to discuss DRPs concerning drug selection, compliance, monitoring and education. Based on our results, it may be considered to only perform MR with the patient when a drug is started and/or a DRP is discussed. Nevertheless, physicians should keep in mind that, if they use information from the EMR and NRMS, unverified dispensing data of the NMRS often contain too much drug information (e.g. stopped drugs). This potentially results in an inappropriate/unnecessary therapy monitoring or withholding of therapy (based on no longer existing interactions) and an increased chance of unnecessary drug safety alerts [43, 44]. Awareness of these aspects is important and the physician should check the actual drug use by the patient in case of a questionable situation.

Strength of our study is that the required drug information to perform drug-related actions was assessed by both individual rheumatologists and an expert panel. We observed discrepancies between the assessments of the rheumatologists and the expert panel: rheumatologists had a lower need for drug information to perform drug-related actions during the visits compared to the expert panel. This could be explained by several causes. First, the need for information was based on a doctor’s individual opinion, and therefore unmeasured subconscious decisions could not be taken into account. Second, there may have been a time constraint during the interviews with the individual rheumatologists after the visits which resulted in underreporting of required drug information. Third, due to time constraints also not all observed drug-related actions were discussed with the rheumatologists by the observer. As a result it was not known for all actions whether the rheumatologist needed information to perform the drug-related action. Fourth, the expert panel also included two pharmacists who had additional drug knowledge compared to rheumatologists which may have affected the joint assessment. As the assessment of the expert panel was subjected to fewer limitations compared to the individual rheumatologists, we assume that the added value of patient’s input during MR based on the assessment of the expert panel gives a more objective representation.

This study has several limitations. First, the rheumatologists who conducted the outpatient visits were not blinded about the aim of the study. Before the outpatient visit, the observer asked the rheumatologist to look at the BPMH. So rheumatologists were urged to use the BPMH, whereas in daily practice it is possible that the BPMH is not always used during the outpatient visit by rheumatologists. This might have led to an overestimation of the added value of medication reconciliation for creating the BPMH in clinical practice.

Second, it is possible that the rheumatologists had a greater focus on medication because they were observed by a pharmacy technician. This potentially resulted in more drug-related actions and increased the chance of overestimating the added value of performing MR in clinical practice. However, the presence of an observer during the visit may have been a barrier for patients to discuss DRPs with the rheumatologist, which may have resulted in an underestimation of the number of discussed DRPs.

Third, there is a chance of recall bias by the rheumatologists of the expert panel, because these rheumatologists also performed 20% of the visits which were observed by the trained pharmacy technician. However, the chance of recall bias is limited because the data were anonymized and there was a large period between performing the observations and consulting the expert panel (2018 versus 2020). Besides that, the assessment made by the (four members of the) expert panel may be different compared to the assessment made by the individual rheumatologist because the expert panel discussed until consensus was achieved.

Fourth, the external validity of this research might be limited because the research was performed with a specific population, in a single hospital, and only in one type of department. Possibly, patient characteristics have influenced MR outcome. The median number of drugs in our population was lower compared to other outpatient clinics like the cardiology or geriatric outpatient clinics [27, 45, 46]. Besides that, it is possible that patients visiting the rheumatology clinic have a higher medication adherence compared to patients with less symptomatic diseases due to the direct (pain-relieving) effects of the used medication. This may have resulted in a lower added value of outpatient MR in our specific population. However, interventions and medications used in rheumatology are comparable to other autoimmune diseases like inflammatory bowel disease or psoriasis. Therefore, our results may still be applicable to more outpatient settings.

Fifth, the results of this study rely to a large extent on the quality and process of creating the BPMH, EMR and NRMS. So, further research is required to confirm our results in other/larger outpatient clinics. However, we expected that the results of the EMR are also generalizable to other countries (without access to the NRMS) as the EMR includes the electronic prescribing system and the physician’s medical notes which are mostly available in other countries.

## Conclusions

Altogether, we observed that in at most one third of outpatient rheumatology visits, patients provided additional information during MR that was required for the rheumatologist to perform drug-related actions when they only had access to the EMR. However, if both the EMR and NMRS were available, patient’s additional input during MR for rheumatologists to perform drug-related actions was less. The added value of patient’s input during MR is highest when a new drug is started or DRPs are discussed. Consequently, to achieve a more targeted and cost-effective implementation of MR, it could be recommended that MR is only performed (during the visit) on these specific occasions. In addition to this, patients could be stimulated to use a personal health record for MR, decreasing the need for pharmacy technicians in this process. However, before these recommendations can be applied in practice, further research is required to confirm our results in other outpatient clinics.

## Supplementary Information

**Additional file 1.** Data collection form.pdf. This document shows the data collection form used during the research.

**Additional file 2.** Assessment form for the expert panel.pdf. This document shows the assessment form used by the expert panel.

**Additional file 3.** Items of the assessment form of the expert panel where consensus was reached.pdf. This document shows the nine items of the assessment form were consensus was achieved by the expert panel.

## Data Availability

The datasets used and/or analysed during the current study are available from the corresponding author on reasonable request.

## Abbreviations

BPMH: Best possible medication history
DRPs: Drug-related problems
EMR: Electronical medical record
IQR: Interquartile range
MR: Medication reconciliation
NMRS: Nationwide Medication Record System
SD: Standard deviation
