# Supplementary material for: The value of incorporating patient-consulted medication reconciliation in influencing drug-related actions in the outpatient rheumatology setting
Source: BMC Health Serv Res. 2022 Aug 5;22:995. doi: 10.1186/s12913-022-08391-7 (PMC9354341; doi:10.1186/s12913-022-08391-7)
Supplement: Supplementary file 1 — Additional file 1. Data collection form.pdf. This document shows the data collection form used during the research. [file 12913_2022_8391_MOESM1_ESM.pdf]

Additionale file 1: Data collection form

Date: ……-……-…………

Patient number reported in HIX: …………….

Informed consent: Yes/No

**General description of the consultation**		
**Drug-related action(s)**	**Used drug information:**<br>**Presence (P)**<br>**Strength (S)**<br>**Frequency (F)**<br>**Drug not in use (N)**	**Explanation**
	P:<br>S:<br>F:<br>N:	
	P:<br>S:<br>F:<br>N:	
**Additional questions asked by the rheumatologists to verify patient's drug use**		
