# Supplementary material for: The value of incorporating patient-consulted medication reconciliation in influencing drug-related actions in the outpatient rheumatology setting
Source: BMC Health Serv Res. 2022 Aug 5;22:995. doi: 10.1186/s12913-022-08391-7 (PMC9354341; doi:10.1186/s12913-022-08391-7)
Supplement: Supplementary file 2 — Additional file 2. Assessment form for the expert panel.pdf. This document shows the assessment form used by the expert panel. [file 12913_2022_8391_MOESM2_ESM.pdf]

Additional file 2: Assessment form for the expert panel

The left column of the table shows drug-related actions which can be performed by a rheumatologist during an outpatient visit. This concern changing current drugs (start, stop and change the frequency or strength of a current drug) or discuss drug-related problems.

Could you indicate which information (listed in column 2) is required to perform each drug-related action? Cases which are exceptions of these general assessment, will be discussed later.

**Drug-related action**	**Required information:**
**Start a drug**	○ Knowledge of all drugs used by the patient? Yes / No
**Restart a drug**	○ Knowledge that the drug was stopped? Yes / No<br>○ Knowledge of all drugs used by the patient, if:<br>○ Duration unknown or > ….. weeks → Yes<br>○ Duration < ….. weeks → No
**Stop a drug**	○ Usage of the concerning drug? Yes / No
**Increase the strength of a drug**	○ Usage of the concerning drug? Yes / No<br>○ The strength of the concerning drug? Yes / No<br>○ The frequency of the concerning drug? Yes / No
**Decrease the strength of a drug**	○ Usage of the concerning drug? Yes / No<br>○ The strength of the concerning drug? Yes / No<br>○ The frequency of the concerning drug? Yes / No
**Increase the frequency of a drug**	○ Usage of the concerning drug? Yes / No<br>○ The strength of the concerning drug? Yes / No<br>○ The frequency of the concerning drug? Yes / No
**Decrease the frequency of a drug**	○ Usage of the concerning drug? Yes / No<br>○ The strength of the concerning drug? Yes / No<br>○ The frequency of the concerning drug? Yes / No
**Drug-related problem: the patient decided to stop using a drug**	○ Information that the drug was stopped (by the patient)? Yes / No
**Drug-related problem: the patient uses a drug other than prescribed**	○ Usage of the concerning drug? Yes / No<br>○ The strength of the concerning drug? Yes / No<br>○ The frequency of the concerning drug? Yes / No
**Drug-related problem: the patient has a problem with intake or**	○ Usage of the concerning drug? Yes / No<br>○ The strength of the concerning drug? Yes / No

**administration of a drug**	○ The frequency of the concerning drug? Yes / No
**Drug-related problem: the patient worried about a specific drug**	○ Usage of the concerning drug? Yes / No
**Drug-related problem: the patient worried about his/her drug or adverse drug reactions of their drugs in general**	○ Usage of all drugs used by the patient? Yes / No
**Drug-related problem: the used drug is not effective**	○ Usage of the concerning drug? Yes / No<br>○ The strength of the concerning drug? Yes / No<br>○ The frequency of the concerning drug? Yes / No
**Injection administrated during the outpatient visit**	○ Usage of the concerning drug? Yes / No
