# Supplementary material for: The value of incorporating patient-consulted medication reconciliation in influencing drug-related actions in the outpatient rheumatology setting
Source: BMC Health Serv Res. 2022 Aug 5;22:995. doi: 10.1186/s12913-022-08391-7 (PMC9354341; doi:10.1186/s12913-022-08391-7)
Supplement: Supplementary file 3 — Additional file 3. Items of the assessment form of the expert panel where consensus was reached.pdf. This document shows the nine items of the assessment form were consensus was achieved by the expert panel. [file 12913_2022_8391_MOESM3_ESM.pdf]

Additional file 3: Items of the assessment form of the expert panel where consensus was reached

The left column of the table shows drug-related actions which can be performed by a rheumatologist during an outpatient visit. This concern changing current drugs (start, stop and change the frequency or strength of a current drug) or discuss drug-related problems.

The right column shows the required information to perform each drug-related action. The answers which were reported by each of the four members of the expert panel are filled in.

Drug-related action	Required information:
**Start a drug**	○ Knowledge of all drugs used by the patient? Yes / ~~No~~
**Stop a drug**	○ Usage of the concerning drug? Yes / ~~No~~
**Increase the strength of a drug**	○ Usage of the concerning drug? Yes / ~~No~~<br>○ The strength of the concerning drug? Yes / ~~No~~<br>○ The frequency of the concerning drug? Yes / ~~No~~
**Decrease the strength of a drug**	○ Usage of the concerning drug? Yes / ~~No~~<br>○ The strength of the concerning drug? Yes / ~~No~~<br>○ The frequency of the concerning drug? Yes / ~~No~~
**Increase the frequency of a drug**	○ Usage of the concerning drug? Yes / ~~No~~<br>○ The strength of the concerning drug? Yes / ~~No~~<br>○ The frequency of the concerning drug? Yes / ~~No~~
**Decrease the frequency of a drug**	○ Usage of the concerning drug? Yes / ~~No~~<br>○ The strength of the concerning drug? Yes / ~~No~~<br>○ The frequency of the concerning drug? Yes / ~~No~~
**Drug-related problem: the patient decided to stop using a drug**	○ Information that the drug was stopped (by the patient)? Yes / ~~No~~
**Drug-related problem: the patient uses a drug other than prescribed**	○ Usage of the concerning drug? Yes / ~~No~~<br>○ The strength of the concerning drug? Yes / ~~No~~<br>○ The frequency of the concerning drug? Yes / ~~No~~
**Drug-related problem: the used drug is not effective**	○ Usage of the concerning drug? Yes / ~~No~~<br>○ The strength of the concerning drug? Yes / ~~No~~<br>○ The frequency of the concerning drug? Yes / ~~No~~
